# The impact of the rs8005161 polymorphism on G protein-coupled receptor GPR65 (TDAG8) pH-associated activation in intestinal inflammation

**DOI:** 10.1186/s12876-018-0922-8

**Published:** 2019-01-07

**Authors:** Irina V. Tcymbarevich, Jyrki J. Eloranta, Jean-Benoît Rossel, Nicole Obialo, Marianne Spalinger, Jesus Cosin-Roger, Silvia Lang, Gerd A. Kullak-Ublick, Carsten A. Wagner, Michael Scharl, Klaus Seuwen, Pedro A. Ruiz, Gerhard Rogler, Cheryl de Vallière, Benjamin Misselwitz, on behalf of the Swiss IBD Cohort Study Group, Karim Abdelrahman, Gentiana Ademi, Patrick Aepli, Amman Thomas, Claudia Anderegg, Anca-Teodora Antonino, Eva Archanioti, Eviano Arrigoni, Diana Bakker de Jong, Bruno Balsiger, Polat Bastürk, Peter Bauerfeind, Andrea Becocci, Dominique Belli, José M. Bengoa, Luc Biedermann, Janek Binek, Mirjam Blattmann, Stephan Boehm, Tujana Boldanova, Jan Borovicka, Christian P. Braegger, Stephan Brand, Lukas Brügger, Simon Brunner, Patrick Bühr, Bernard Burnand, Sabine Burk, Emanuel Burri, Sophie Buyse, Dahlia-Thao Cao, Ove Carstens, Dominique H. Criblez, Sophie Cunningham, Fabrizia D’Angelo, Philippe de Saussure, Lukas Degen, Joakim Delarive, Christopher Doerig, Barbara Dora, Susan Drerup, Mara Egger, Ali El-Wafa, Matthias Engelmann, Jessica Ezri, Christian Felley, Markus Fliegner, Nicolas Fournier, Montserrat Fraga, Yannick Franc, Pascal Frei, Remus Frei, Michael Fried, Florian Froehlich, Raoul Ivano Furlano, Luca Garzoni, Martin Geyer, Laurent Girard, Marc Girardin, Delphine Golay, Ignaz Good, Ulrike Graf Bigler, Beat Gysi, Johannes Haarer, Marcel Halama, Janine Haldemann, Pius Heer, Benjamin Heimgartner, Beat Helbling, Peter Hengstler, Denise Herzog, Cyrill Hess, Roxane Hessler, Klaas Heyland, Thomas Hinterleitner, Claudia Hirschi, Petr Hruz, Pascal Juillerat, Carolina Khalid-de Bakker, Stephan Kayser, Céline Keller, Christina Knellwolf, Christoph Knoblauch, Henrik Köhler, Rebekka Koller, Claudia Krieger, Patrizia Künzler, Rachel Kusche, Frank Serge Lehmann, Andrew Macpherson, Michel H. Maillard, Michael Manz, Astrid Marot, Rémy Meier, Christa Meyenberger, Pamela Meyer, Pierre Michetti, Benjamin Misselwitz, Patrick Mosler, Christian Mottet, Christoph Müller, Beat Müllhaupt, Leilla Musso, Michaela Neagu, Cristina Nichita, Jan Niess, Andreas Nydegger, Nicole Obialo, Diana Ollo, Cassandra Oropesa, Ulrich Peter, Daniel Peternac, Laetitia Marie Petit, Valérie Pittet, Daniel Pohl, Marc Porzner, Claudia Preissler, Nadia Raschle, Ronald Rentsch, Alexandre Restellini, Sophie Restellini, Jean-Pierre Richterich, Frederic Ris, Branislav Risti, Marc Alain Ritz, Gerhard Rogler, Nina Röhrich, Jean-Benoît Rossel, Vanessa Rueger, Monica Rusticeanu, Markus Sagmeister, Gaby Saner, Bernhard Sauter, Mikael Sawatzki, Michael Scharl, Martin Schelling, Susanne Schibli, Hugo Schlauri, Dominique Schluckebier, Daniela Schmid, Sybille Schmid, Jean-François Schnegg, Alain Schoepfer, Vivianne Seematter, Frank Seibold, Mariam Seirafi, Gian-Marco Semadeni, Arne Senning, Christiane Sokollik, Joachim Sommer, Johannes Spalinger, Holger Spangenberger, Philippe Stadler, Peter Staub, Dominic Staudenmann, Volker Stenz, Michael Steuerwald, Alex Straumann, Bruno Strebel, Andreas Stulz, Michael Sulz, Aurora Tatu, Michela Tempia-Caliera, Joël Thorens, Kaspar Truninger, Radu Tutuian, Patrick Urfer, Stephan Vavricka, Francesco Viani, Jürg Vögtlin, Roland Von Känel, Dominique Vouillamoz, Rachel Vulliamy, Paul Wiesel, Reiner Wiest, Stefanie Wöhrle, Samuel Zamora, Silvan Zander, Tina Wylie, Jonas Zeitz, Dorothee Zimmermann

**Affiliations:** 1 Department of Gastroenterology and Hepatology, University Hospital Zurich, University of Zurich, Zurich, Switzerland; 2 Clinical Pharmacology and Toxicology, University Hospital Zurich, University of Zurich, Zurich, Switzerland; 3 Insitute of Social and Preventative Medicine, Lausanne, Switzerland; 4 Institute of Physiology, University of Zurich, Zurich, Switzerland; 5 Novartis Institutes for Biomedical Research, Basel, Switzerland; 6 Present address: Department of Viceral Surgery and Medicine, Inselspital Bern and University of Bern, Freiburgstr. 18, 3010 Bern, Switzerland

**Keywords:** pH-sensing, RhoA, Acidic pH, cAMP, Inflammatory bowel diseases, IBD, CD, UC

## Abstract

**Background:**

Tissue inflammation in inflammatory bowel diseases (IBD) is associated with a decrease in local pH. The gene encoding G-protein-coupled receptor 65 (GPR65) has recently been reported to be a genetic risk factor for IBD. In response to extracellular acidification, proton activation of GPR65 stimulates cAMP and Rho signalling pathways. We aimed to analyse the clinical and functional relevance of the GPR65 associated single nucleotide polymorphism (SNP) rs8005161.

**Methods:**

1138 individuals from a mixed cohort of IBD patients and healthy volunteers were genotyped for SNPs associated with GPR65 (rs8005161, rs3742704) and galactosylceramidase (rs1805078) by Taqman SNP assays. 2300 patients from the Swiss IBD Cohort Study (SIBDC) were genotyped for rs8005161 by mass spectrometry based SNP genotyping. IBD patients from the SIBDC carrying rs8005161 TT, CT, CC and non-IBD controls (CC) were recruited for functional studies. Human CD14+ cells were isolated from blood samples and subjected to an extracellular acidic pH shift, cAMP accumulation and RhoA activation were measured.

**Results:**

In our mixed cohort, but not in SIBDC patients, the minor variant rs8005161 was significantly associated with UC. In SIBDC patients, we observed a consistent trend in increased disease severity in patients carrying the rs8005161-TT and rs8005161-CT alleles. No significant differences were observed in the pH associated activation of cAMP production between IBD (TT, CT, WT/CC) and non-IBD (WT/CC) genotype carriers upon an acidic extracellular pH shift. However, we observed significantly impaired RhoA activation after an extracellular acidic pH shift in IBD patients, irrespective of the rs8005161 allele.

**Conclusions:**

The T allele of rs8005161 might confer a more severe disease course in IBD patients. Human monocytes from IBD patients showed impaired pH associated RhoA activation upon an acidic pH shift.

**Electronic supplementary material:**

The online version of this article (10.1186/s12876-018-0922-8) contains supplementary material, which is available to authorized users.

## Background

Inflammatory bowel disease (IBD) is characterized by chronic inflammation of the intestinal tract. It is widely accepted that it is a multifactorial inflammatory disease determined by an interaction between genetic and environmental triggers. Inflammation is associated with an increase in local proton concentration and lactate production [1], with subsequent pro-inflammatory cytokine production. An acidic environment may affect the progression and resolution of inflammation [2–4]. To maintain pH homeostasis, cells are required to sense acidic changes in their environment and respond accordingly. Recently, three G protein-coupled receptors (GPCR), T cell death-associated gene 8 (TDAG8 also known as GPR65), ovarian cancer G protein-coupled receptor 1 (OGR1 also known as GPR68) and G protein coupled receptor 4 (GPR4), have been shown to sense extracellular protons and stimulate a variety of signalling pathways [5–9]. Accumulating evidence indicates that these particular proton-sensing receptors play a crucial role in pH homeostasis [6, 8, 10–12].

Studies in IBD genetics have identified more than 240 regions in the human genome that increase the risk of IBD [13–16]. The majority of IBD specific single nucleotide polymorphisms (SNPs) confer an increased risk for both CD and UC [14]. Some of the CD specific genes are associated with bacterial response genes (e.g. NOD2 and autophagy), while for UC there are several specific genes that are associated with immune response and barrier function [17]. More than 70% of the IBD-risk loci are shared with other immune-mediated inflammatory diseases [14]. Genome wide association studies (GWAS) have identified a locus within the GPR65 (TDAG8) gene as one of the risk loci associated with CD and UC [13, 14]. GPR65 is highly expressed in spleen, thymus, lymph nodes and peripheral blood leukocytes, suggesting an important immune response function, which in turn plays a crucial role during the pathogenesis of IBD. In response to extracellular acidic pH, GPR65 activates the adenylyl cyclase (AC)/cAMP/Protein Kinase A (PKA) pathway through Gs proteins [5, 18] and the Rho signalling pathway via G_12/13_ [5, 7]. Inflammatory processes in the gut are frequently associated with a decrease in local pH, potentially explaining the contribution of GPR65 to intestinal inflammation in IBD.

Determining how genetic polymorphisms can affect functionality is currently a major challenge [19]. In this study, we aimed to test the hypothesis that genetic polymorphisms lead to an altered activity of GPR65, which may result in a higher risk for gut inflammation and IBD. We found that the GPR65 rs8005161 polymorphism has a significant association with UC. Moreover, RhoA activation in human macrophages was significantly lower in acidic conditions for IBD patients vs. non-IBD group.

## Methods

### Study subjects

The study population for the Taqman SNP assay included 591 healthy subjects and 547 IBD patients [20, 21]. All subjects provided written informed consent to be included in the study.

A second cohort was obtained from the Swiss Inflammatory Bowel Disease Cohort Study (SIBDCS), which includes patients with IBD from all regions of Switzerland since 2006 [22]. The cohort goals and methodology are described elsewhere [22]. We included 2300 adult IBD patients that were enrolled in the study and previously genotyped for the risk variant rs8005161 within the GPR65/GALC gene locus. Genotyping was performed as part of an analysis of the whole Swiss IBD cohort for all SNPs that were known to be associated with IBD at that point in time [14]. Patients with IBD were recruited at the centres participating in SIBDCS [22]. Genotyping of SIBDCS patients was performed using MALDI-TOFF mass spectrometry based SNP genotyping [23].

Eight SIBDC patients carrying rs8005161-CC, 9 SIBDC patients carrying rs8005161-CT and 9 SIBDC patients carrying rs8005161-TT provided blood samples for cAMP and RhoA assays. Demographic and clinical data were obtained at the time of the blood collection. Ten healthy volunteers were recruited as controls. All controls were rs8005161-CC. One individual in the control group used nonsteroidal anti-inflammatory drugs (NSAIDs) in low dosage on a regular basis. For this individual, cAMP values were in the medium range of the healthy volunteers and RhoA values could not be determined due to low cell numbers.

### Isolation of CD14+ human peripheral blood monocytes

Human peripheral blood mononuclear cells (PBMCs) were isolated by density gradient centrifugation (Ficoll Histopaque 10,771 SIGMA, USA) and cryopreserved in foetal calf serum (FCS, Gibco, Thermo Fisher Scientific) supplemented with 10% dimethyl sulfoxide. Upon thawing, cell purification was then performed using EasySep™ Human Monocyte CD14 Enrichment Kit (17,858, Stemcell, Canada) according to the manufacturer’s instructions. Monocytes purity was > 85% as assessed by allophycocyanin (APC)-labelled anti-CD14 (#17–0149-42, eBioscience, USA) and Pacific Blue (PB)-labelled anti-CD45 (#304022, Biolegend, USA) by flow cytometry (Additional file 1: Figure S1).

### Genomic DNA extraction

Genomic DNA was isolated from EDTA-blood or intestinal biopsies using the QIAamp DNA Mini Kit (QIAGEN, Hombrechtikon, Switzerland), or QIAzol (Qiagen), respectively, according to manufacturer’s instructions. The concentrations of genomic DNA were quantified using a NanoDrop ND-1000 spectrophotometer (NanoDrop Technologies, Germany).

### Genotyping

Genotyping of SNPs was performed using TaqMan allelic discrimination assays (TaqMan SNP Genotyping Assays C_1928636_10, C_1928640_1_ and C_11667238_10 for the SNPs rs8005161, rs3742704 and rs1805078, respectively, all from Applied Biosystems, Thermo Fisher Scientific, USA) on a 7900HT Fast Real-Time PCR instrument (Applied Biosystems, Rotkreuz, Switzerland) using the following cycling conditions: 10 min at 95 °C, 45 cycles of 95 °C for 15 s, and 60 °C for 1 min. Ten nanograms of each genomic DNA was used per PCR reaction in a volume of 5 μl.

The presence of either major or minor alleles of each subject participating in the cAMP and RhoA functional GPR65 assays was confirmed by Taqman genotyping (rs8005161, C_1928636_10 TaqMan SNP Genotyping Assay).

### pH experiments

Monocytes were subjected to an extracellular acidic shift from pH 7.6, where pH-sensing GPR65 receptor is minimally active or almost silent, to pH 6.6, where it is maximally active. Cells treated at pH 7.6 served as negative controls. pH shift experiments measuring cAMP production were carried out in Hank’s Balanced Salt Solution (HBSS, 14065056, Gibco) with 25 mM HEPES (Gibco) in a 37 °C incubator without CO_2_. For the measurement of RhoA activation, pH shift experiments were carried out in serum free RPMI 1640 medium containing a bicarbonate buffer, supplemented with 2 mM Glutamax (35050–038, Gibco) and 25 mM HEPES. The pH of all solutions was adjusted using a calibrated pH meter (Metrohm, Herisau, Switzerland) by the addition of appropriate quantities of NaOH or HCl. Because the pH adjusted RPMI medium also contains bicarbonate buffer, we allowed the media to equilibrate in a 5% CO_2_ incubator for at least 36 h before use. Control experiments confirmed that the media pH was stable for at least one month under these conditions. The pH was checked before each experiment and found to be stable within a very narrow range (+/− 0.03). RhoA activity assays were incubated in a 5% CO_2_ humidified 37 °C incubator. All data presented in this paper are referenced to pH measured at room temperature.

### cAMP determination

cAMP accumulation following the activation of GPR65 by acidic pH was measured by a competitive cell-based sandwich immunoassay and quantified by homogenous time-resolved fluorescence (HTRF) technology (cAMP Dynamic 62AM4PEC, CisBio, France). Human CD14+ monocytes were seeded at non-activating pH 7.6 HBSS supplemented with 25 mM HEPES in 384-well plates (Cat. No. 781080, Greiner) at 10,000 cells/well in HBSS at pH 7.6 with or without the GPR65 antagonist (10uM) (provided by Novartis Institutes for Biomedical Research, Switzerland), and incubated for 15 min, followed by a 30 min pH shift, which was achieved by addition of the appropriate amount of HBSS buffer to obtain the desired final pH (pH 7.6 or pH 6.5). All incubations were carried out in a non-CO_2_ incubator at 37 °C. The optimal activating pH for GPR65/cAMP –mediated signalling was determined in extensive pH dose response experiments (Additional file 2: Figure S2), and described in detail in the following section. Phosphodiesterase inhibitors (1 mM IBMX, 10 μM Rolipram, 1 μM BAY) were used in all conditions. Samples and the cAMP standards were analysed using a sigmoidal dose response model with variable slope in using the software package GraphPad Prism, La Jolla California USA, www.graphpad.com (San Diego, CA, USA).

### cAMP activation assay validation

Proton-activated GPR65/cAMP –mediated signalling was tested using the human monocytic cell line THP-1 and CD14+ primary human monocytes isolated from non-IBD subjects (WT/CC genotype). To confirm that pH was associated with GPR65/cAMP –mediated signalling, pH dose response experiments were performed. Cells were starved at pH 7.6 for 2 h and subsequently subjected to a pH shift for 10 min (pH 6.2 to 7.8 with 0.2 increments). The highest cAMP accumulation was observed at pH 6.4–6.8, whereas only low cAMP concentrations were observed at pH 7.6–7.8 (Additional file 2: Figure S2). Maximum activation and inactivation of GPR65 were achieved at pH 6.5 and pH 7.6, respectively.

### RhoA GTPase activation assay

Human CD14+ cells were plated in RPMI plus 10% FCS and incubated for 1 h, followed by 2 h of starvation at pH 7.6 in serum free RPMI. The pH shift was performed for 10 min at pH 6.6, with the negative control (pH 7.6). All incubations were carried out in a 5% CO_2_ humidified 37 °C incubator. The pH range was established in pH dose response experiments (Additional file 3: Figure S3). 15 μg of protein was loaded per well and GTP-bound RhoA protein levels were measured in duplicates according to the manufacturer’s instructions (# BK124, Cytoskeleton, USA). Final absorbance (OD_490_) was measured in a Synergy 2 micro-plate reader (Biotek, Luzern, Switzerland). No internal standard for RhoA has been established, therefore baseline values for RhoA cannot be compared between experiments and only normalized values are presented.

### RhoA activation assay validation

To confirm that RhoA activation is proton dependent, THP-1 cells and CD14+ monocytes were subjected to a pH shift (10 min) at different pH levels, with a preliminary starvation step (2 h) at non-activating pH (pH 7.6). As shown in Additional file 3: Figure S3, pH 6.6 elicited a significant increase in RhoA activation compared to pH 6.2, 7.4 and 7.6, which, in contrast, induced no significant activation in CD14+ monocytes and THP-1 cells. Since GPR65/G12/13/RhoA signalling was highest at pH 6.6, this pH was used in all RhoA activity assays.

### Normalization of RhoA and cAMP levels in IBD patients and controls

cAMP and RhoA activity was measured at pH 6.5 and 6.6, respectively and all levels were normalized to RhoA and cAMP levels from the same participant tested at conditions with lowest RhoA and cAMP production. This residual activity (at non-activating pH and in the presence of the inhibitor) is unlikely to be due to GPR65. For cAMP, we normalized to levels at pH 7.6 in the presence of a GPR65 inhibitor. For RhoA activity we normalized to levels at pH 7.6. Due to a high demand of cells from human patients for the RhoA assay, the control at pH 7.6 with the GPR65 inhibitor was not feasible.

### Statistical analysis

Clinical data were retrieved from the data centre of the SIBDCS at the Lausanne University Hospital. These data were entered into a database (Access 2000; Microsoft Switzerland Ltd., Liab., Co., Wallisellen, Switzerland). The Statistical Package for the Social Sciences (version 21; SPSS, Chicago, Ill., USA), GraphPad Prism, version 7, or Stata software (StataCorp., 2015. Stata Statistical Software: Release 14. College Station, TX, USA) was used for the statistical analysis.

The Chi-square test or Fisher’s exact test were used to determine associations between individual SNPs and subject phenotypes. Groups of data were compared using Kruskal-Wallis test, Fisher’s exact, student t test or one-way ANOVA. Data are presented as mean and interquartile range (IQR). Probabilities (*p*, two tailed) of *p* < 0.05 were considered statistically significant. Throughout this manuscript, asterisks denote significant differences at *, *p* < 0.05; **, *p* < 0.01; ***, *p* < 0.001; ****, *p* < 0.0001.

## Results

### Genotyping for rs8005161, rs3742704 and rs1805078 in IBD vs. non-IBD patients by Taqman SNP assays

Genomic DNA from a group of 547 patients with IBD (mean age 55.7 years, range 20–81) and 591 non-IBD subjects (mean age 42.6 years, range 16–82) was used to genotype individuals for genetic variants rs8005161 [13, 14], rs3742704 [12, 13] (both assigned to the GPR65 gene), and an additional neighbouring SNP rs1805078 (galactosylceramidase, GALC). Frequencies of homozygous and heterozygous carriers of the major and minor alleles, respectively are provided in Table 1. For rs8005161 (GPR65), the frequency of individuals homozygous for the minor T allele was significantly higher in UC individuals compared to individuals without IBD (p < 0.05). When comparing the number of individuals carrying at least one T allele (i.e. CT + TT vs. CC) we noticed a trend for higher frequency of the T carriers in UC patients which did not reach significance. In contrast, in individuals with CD the frequency of rs8005161 alleles did not differ from non-IBD carriers. There were no significant differences in the genotype and allele frequencies distribution between the groups for the GPR65 variant rs3742704 and the GALC variant rs1805078. In a complementary analysis, we compared the total number of rs8005161 minor alleles which differed significantly between UC patients and healthy volunteers but not when CD patients or the other SNPs were evaluated (Additional file 4: Table S1).

**Table 1:** Genotype frequencies and genotype association analysis of mixed population of IBD patients and healthy subjects

				Minor allele homozygous carriers		Minor allele carriers			
rs8005161, GPR65									
	CC (%)	CT (%)	TT (%)	p-value	OR (CI)	*p*-value	OR (CI)	*Χ* ^*2*^ *(HWE) p-value*	
non-IBD	422 (80.8%)	95 (18.2%)	5 (1.0%)						
IBD	277 (76.5%)	78 (21.6%)	7 (1.9%)	0.25	2.04 (0.64–6.5)	0.13	1.3 (0.93–1.8)		0.2
UC	105 (74.5%)	31 (21.9%)	5 (3.6%)	**0.04**	**3.8 (1.09–13)**	0.1	1.45 (0.93–2.2)		**0.04**
CD	172 (77.8%)	47 (21.3%)	2 (0.9%)	1	0.94 (0.18–4.9)	0.37	1.2 (0.82–1.8)		0.62
rs3742704, GPR65									
	AA (%)	AC (%)	CC (%)	p-value	OR (CI)	*p*-value	OR (CI)		*Χ* ^*2*^ *(HWE)p-value*
non-IBD	317 (84.3%)	57 (15.2%)	2 (0.5%)						
IBD	343 (80.3%)	78 (18.3%)	6 (1.4%)	0.29	2.7 (0.53–13)	0.17	1.3 (0.91–1.9)		0.21
UC	99 (81.1%)	23 (18.9%)	0	1	0.61 (0.03–13)	0.4	1.3 (0.73–2.1)		0.46
CD	239 (80.2%)	54 (18.1%)	5 (1.7%)	0.25	3.2 (0.61–17)	0.18	1.3 (0.89–2)		0.19
rs1805078, GALC									
	GG (%)	GA (%)	AA (%)	p-value	OR (CI)	*p*-value	OR (CI)		*Χ* ^*2*^ *(HWE) p-value*
non-IBD	270 (88.2%)	33 (10.8%)	3 (1%)						
IBD	363 (88.1%)	47 (11.4%)	2 (0.5%)	1	0.49 (0.082–3)	1	1.01 (0.64–1.6)		0.71
UC	116 (86.6%)	15 (11.2%)	3 (2.2%)	0.37	2.3 (0.46–11.6)	0.63	1.6 (0.63–2.1)		0.570
CD	242 (88.6%)	31 (11.4%)	0	0.25	0.16 (0.008–3.1)	0.9	0.96 (0.58–1.6)		1

Odds ratio (OR) with 95% confidence interval (CI) and *p*–value for WT vs. homozygous and heterozygous allele carriers is indicated. Statistical analysis: Fisher’s exact test with two-tailed values; HWE – calculated *χ*^*2*^ for Hardy-Weinberg Equilibrium. CD: Crohn’s disease, GALC: galactosylceramidase, GPR65: G protein-coupled receptor 65 (also known as TDAG8), IBD: inflammatory bowel disease, UC: ulcerative colitis

When patients with UC and CD were compared, no difference between individuals carrying at least one T allele (i.e. CT + TT vs. CC) or number of T alleles was found (*p* = 0.52; *p* = 0.25).

### Genotyping of Swiss IBD cohort study (SIBDC) patients for GPR65 IBD risk SNP rs8005161

The SIBDC comprises a data base of clinical and genetic data of patients with IBD. Genotype data for rs8005161 (GPR65) for 2300 SIBDCS patients are available; rs3742704 (GPR65) and rs1805078 (GALC) have not been genotyped in SIBDC. No control cohort is available for SIBDC. For the SNP rs8005161 (major allele: C, minor allele: T), 28/2300 patients (1.2%) were homozygous TT carriers, 430/2300 (18.7%) heterozygous CT carriers, and 1842/2300 (80.1%) were wildtype (WT) CC patients. Allele frequencies for T and C genotype were 10.6% (426) and 89.4% (3710), respectively (Table 2). Overall, carriers of the T allele tended to be diagnosed more often with CD (NS). Thus, the possible association of the T allele of rs8005161 with UC in our initial cohort could not be confirmed with SIBDC data.

**Table 2:** Demographic characteristics and biological phenotypes of carriers of various alles of the GPR65 SNP rs8005161 for patients from the SIBDC

	rs8005161 CC	rs8005161 CT or TT	p-value (Fisher or Kruskal-Wallis)
Diagnosis			
Crohn’s disease	1051 (57.1%)	284 (62.0%)	0.057 (NS)
UC / IC	791 (42.9%)	174 (38.0%)	
Gender	953 (51.7%)	238 (52.3%)	0.83 (NS)
	889 (48.3%)	220 (47.7%)	
Age at diagnosis [years]			
median, (IQR),	26.4, (19.1–36.6),	25.1, (18.2–36.3),	0.059 (NS)
min – max	0.5–81.4	2.6–77.5	
Disease duration [years]			
Median (IQR),	12.2, (7.3–20.5),	12.3, (7.3–20.7),	0.998 (NS)
min – max	0.1–52.4	0.3–56.6	
Last BMI [kg /m^2^]			
median, (IQR),	23.8, (21.1–26.7),	23.2, (20.7–26.1),	**0.020**
min – max	12.6–47.1	13.2–46.3	
Intestinal surgery			
No (*n* = 1620)	1311 (71.2%)	309 (67.5%)	0.123 (NS)
Yes (*n* = 680)	531 (28.8%)	149 (32.5%)	
Past or current therapy with biologics			
No (*n* = 1107)	909 (49.3%)	198 (43.2%)	**0.021**
Yes (*n* = 1193)	933 (50.7%)	260 (56.8%)	

Significant phenotypes are indicated in **bold**

*IQR* interquartile range, *IC* indeterminate colitis, *CD* Crohn’s disease, *UC* ulcerative colitis

Interestingly, we noted a consistent trend for a more severe disease course with an earlier age at diagnosis (NS), a lower body mass index (*p* = 0.02), a higher frequency of surgery (NS) and a higher frequency of therapy with biologics (p = 0.02). A similar trend for body mass index (BMI) and past or current therapy with biologics was confirmed when the CC, CT and TT genotypes were considered separately (Additional file 5: Table S2). However, for intestinal surgery this trend was not confirmed, and likely due to the small size of the TT group.

### pH-dependent activation of cAMP production is not affected by the rs8005161 T-variant in CD14+ cells

Next, we sought to determine whether the GPR65 SNP variant rs8005161 has an impact on GPR65 protein functionality and downstream signalling cascades in vitro, by measuring production of cAMP and activation of RhoA, two well-established second massagers downstream of GPR65. To validate pH-dependent GPR65 activation, we performed pH dose response experiments in a human monocyte cell line (THP-1) and in CD14+ human monocytes, isolated from healthy volunteers (Additional file 1: Figure S1). Our data confirm maximum of cAMP and RhoA activity at pH 6.5 and 6.6, respectively (Additional file 2: Figure S2 and Additional file 3: Figure S3). For subsequent experiments, these pH values were used and normalized to levels from the same participant at conditions with the least activity.

We recruited patients from the SIBDC with TT, CT and CC genotype based on the results from SIBDCS sequencing, in addition to healthy CC volunteers, as described in the Materials and Methods section. Patient characteristics are shown in Table 3. Patients with different alleles of rs8005161 were of similar age and had a similar distribution of UC and CD; however, in our sample cohort, individuals with the TT genotype had lower disease activity with a lower frequency of TNF-inhibitor or Vedolizumab usage.

**Table 3:** Demographic and clinical data of healthy volunteers and patients with different alleles of GPR65 SNP rs8005161 used for further analysis

	Healthy controls	rs8005161 CC	rs8005161 CT	rs8005161 TT
Number of patients	8	8	9	9
Gender, females	3 (37.5%)	4 (50%)	3 (33%)	6 (66%)
Age (median ± IQR)	42 (37.5–47.5)	46.7 (37.9–50.6)	41.3 (33.9–51.4)	43.1 (27.5–48.8)
Diagnosis				
UC/ CD (percent UC)	NA	4/4 (50%)	6/3 (66%)	6/3 (66%)
Disease severity				
Harvey-Bradshaw Index (median, IQR)	NA	6.0 (4.3–7.5)	4 (3–4)	1 (0.5–1)
UC Severity Index (median, IQR)	NA	4.0 (3.3–4.8)	7.5 (3.5–10)	0 (0–1.5)
Medical history				
Azathioprine / 6-Mercaptopurine	NA	1/8	1/9	2/8
Methotrexate	NA	0/8	1/9	0/8
Tacrolimus	NA	1/8	0/9	0/8
Tumor necrosis factor inhibitor	NA	4/8	3/9	1/8
Vedolizumab	NA	4/8	5/9	1/8
Systemic steroids ≥10 mg/d	NA	0/8	3/9	1/8
Oral mesalazine/ sulfasalazine	NA	5/8	3/9	4/8

*CD* Crohn’s disease, *IBD* inflammatory bowel disease, *IQR* interquartile range, *NA* not applicable, *UC* ulcerative colitis

**Fig. 1:**
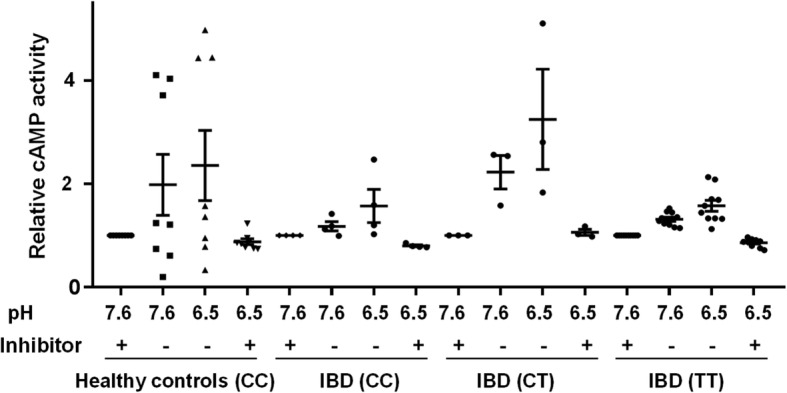
Formation of cAMP in human CD14+ monocytes upon pH shift from pH 7.6 to pH 6.5. 10 μM of G protein-coupled receptor 65 (GPR65) antagonist was used when indicated (+). Human CD14+ cells were obtained from IBD patients carrying either rs8005161 TT, CT or WT/CC genotype, and non-IBD and WT/CC genotype. Produced cAMP was calculated as a ratio of the respective condition relative to pH 7.6 plus inhibitor. No significant differences between the genotypes were identified. cAMP: cyclic adenosine monophosphate, IBD: inflammatory bowel disease, WT: Wild type

As shown in Fig. 1, normalized cAMP level increased in PBMCs from all groups upon an extracellular acidic pH shift from pH 7.6 to pH 6.5. In the presence of the GPR65 antagonist, no activation of cAMP production was observed upon pH shift, confirming that pH-stimulated cAMP production is mediated by GPR65. No difference between individuals with CC, CT and TT genotype could be detected (*p* > 0.05, data not shown), indicating that the genotype variant of GPR65 rs8995161 has no influence on the GPR65/Gs/cAMP pathway in human monocytes under the conditions tested.

We also note differences in the residual cAMP activity in the presence of the GPR65 inhibitor (Additional file 6: Figure S4). Since these differences were observed in the presence of the GPR65 inhibitor at the non-activating pH 7.6, they are unlikely to represent GPR65 activity and the underlying physiology remains unclear.

### pH dependent activation of rho a production is significantly decreased in IBD patients

**Fig. 2:**
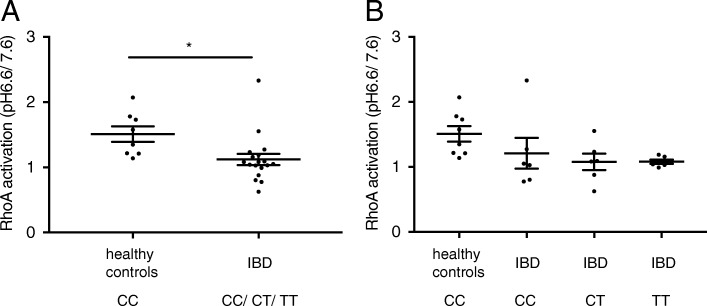
Formation of RhoA in human CD14+ monocytes upon pH shift from pH 7.6 to pH 6.6*.* **a** Activated GTPase RhoA in human CD14+ monocytes upon pH shift from pH 7.6 to pH 6*.*6 (*n* = 6). Carriers of rare TT genotype showed the lowest level of RhoA activation compared to heterozygous CT, WT/CC or healthy WT/CC subjects. **b** Significantly decreased activation of GTPase RhoA in CD14+ monocytes of IBD (WT/CC, CT, TT) patients compared to non-IBD (WT/CC) genotype carriers upon pH shift 7.6 to 6.6. Data points are normalized to pH 7.6 condition (n = 6 non-IBD, *n* = 18 IBD). Each dot represents a single patient, one-way ANOVA, t-test, * *p* < 0.05. No significant differences between carriers of different alleles were identified. IBD: inflammatory bowel disease, WT: Wild type

The GPR65 receptor is also coupled to G_12/13_ proteins, which are known to mediate small GTPase RhoA activation by phosphorylation. Consequently, we next investigated the effects of the GPR65 rs8005161 variant on this signalling pathway. An acidic pH shift from pH 7.6 to pH 6.6 resulted in activation of RhoA. Interestingly, this stimulation was lower for IBD patients compared to the healthy control subjects (*p* < 0.05, Fig. 2a). No differential RhoA GTPase activation in CD14+ monocytes according to the rs8005161 genotype could be detected (Fig. 2b). Experiments with the GPR65 antagonist were not feasible due to the limited amount of human material. Thus, CD14+ monocytes from IBD patients demonstrate reduced RhoA activation upon acidic pH shift compared to healthy control subjects.

## Discussion

In this study, we addressed the clinical relevance of the SNP rs8005161 GPR65 variant as a risk gene for IBD, and tested a potential functional consequence of this SNP variant in human CD14+ monocytes/ macrophages. We found several indications for a more severe clinical phenotype in IBD patients with the T allele of the GPR65 SNP rs8005161 in patients from the SIBDC. We examined proton-activated TDAG8 -mediated signalling pathways in CD14+ monocytes from IBD rs8005161 (WT/CC, CT, TT) and non-IBD (WT/CC) carriers. We observed lower RhoA GTPase activation upon an acidic pH shift in IBD patients compared to healthy volunteers. No differential activation of either RhoA or cAMP stimulated by acidosis was detected in individuals with different rs8005161 genotypes, thus rendering major effects of the CC, CT or TT variants on cAMP or RhoA activation under the conditions tested unlikely.

The GPR65 gene encodes a transmembrane receptor, which is reported to function as a proton sensor [5, 18, 24, 25]. Previously, psychosine (1-β-D-galactosylsphingosine) was proposed to activate GPR65 [26], however the study showed no data for the specific interaction of psychosine with GPR65, and subsequent reports have not supported this finding [5, 18, 24, 25]. Recently, BTB09089 (3-[(2,4-dichlorobenzyl)thio]-1,6-dimethyl-5,6-dihydro-1H-pyridazino[4,5 e][1,3,4]thiadiazin-5-one) has been reported to be an allosteric modulator for GPR65 [27]. However, despite numerous studies, the physiological role of GPR65 under inflammatory conditions (acidic pH) is unclear. GPR65 is mainly expressed in immune cells [28, 29], suggesting an immunological role. Proton activation of GPR65 stimulates second messengers Gs/cAMP and G_12/13_/RhoA [5, 7, 30]. Second messenger cAMP is produced by the activation of adenylyl cyclase, which converts adenosine triphosphate (ATP) into the biologically active signalling mediator, with subsequent degradation by phosphodiesterases (PDEs). The role of cAMP in regulating inflammatory diseases has been well studied [31, 32] and there is continued interest in cAMP as a therapeutic target to treat inflammatory diseases [33]. Increased cAMP levels can inhibit the secretion of proinflammatory cytokines and chemokines [27, 30, 34–37], inhibit inflammatory cell migration [38–40] and modulate epithelial barrier formation [41–43]. In the present study, we observed TDAG8–mediated increases in cAMP at reduced pH in peripheral monocytes. We could confirm TDAG8 signalling by using a specific TDAG8 inhibitor. However, no differences between IBD variants and non-IBD subjects were detected, suggesting that major effects of cAMP signalling at the conditions tested unlikely.

There is increasing interest in small GTPases of the Rho family as candidates for therapeutic intervention due to their involvement in a wide variety of diseases. Rho functions as a molecular switch, in the GTP-bound conformation, the proteins are able to interact with their downstream targets and transmit signals to the cell [44]. Rho GTPases are critical regulators of many cellular functions including cytoskeletal remodelling, cell-cell adhesion, cell polarization, vesicle trafficking, morphogenesis, apoptosis, cell migration, organelle development, membrane transport pathways and tumour motility and proliferation [44–51]. Our study identified a lower RhoA activation in response to acidic pH from monocytes of IBD patients compared to healthy volunteers, but no differences between the rs8005161 GPR65 CC, CT or TT variants. These data are in line with a role of pH sensing for immune activation in IBD pathogenesis. The reasons for this differential activation remain unclear and possible explanations include a higher baseline activation in IBD patients with (subclinical) disease. Alternatively, cell migration and wound healing may be impaired in IBD patients. Thus, future studies should address this issue with more mechanistic studies.

Our analysis did not identify genotype dependent changes in either RhoA or cAMP activation. There might be several explanations for these findings: i) differential activation might only be relevant in subsets of macrophages or other cell types. ii) differential effects of the genotypes might not be mediated via cAMP or RhoA but other properties of GPR65. One example would be genotype dependent differential inhibition of Rac1. Inhibition of Rac1 via the via G_12/13_ pathway has as described previously [52] and Rac1 inhibition has been associated with remission in IBD [53]. iii) finally, considering small effects of rs8005161 polymorphisms [14] (increase of CD risk by an odds ratio (OR) of 1.16 (1.09–1.22) and UC by an OR of 1.14 (1.08–1.21)) our study with a limited number of participants might be underpowered to detect small differences in the activation of secondary messengers.

Most IBD risk genes increase the risk of both, UC and CD in carriers [14]. In a recent meta-analysis, OR for carriers of rs8005161 for CD was 1.156 (CI: 1.092–1.222), the OR for UC was 1.143 (CI: 1.076–1.213) [14]. In agreement with a general increase in the risk for IBD, no differences between allelic frequencies of rs8005161 between CD and UC patients was found in our study.

Associations of IBD genotypes with subsequent disease course have been difficult to establish. However, disease location in CD (ileal vs. colonic) could be predicted by a compound genetic risk score [54]. As expected, for rs8005161 no association with disease location was observed in agreement with highly similar OR for CD (predicting ileal disease) and UC (predicting colonic disease). Furthermore, genetic predisposition can predict onset of disease and individuals with a high genetic burden developed CD 5 years earlier than individuals with the lowest genetic risk [55]. In agreement with this notion, carriers of the rs8005161 T allele developed disease 1.3 years before rs8005161-CC carriers; however, probably due to the wide distribution of the year of onset of disease, this difference failed to reach statistical significance. Associations of genetic risks and clinical course or treatment of IBD are insufficiently understood; most likely due to limited precision of data recorded in large IBD cohort data bases. Analysis of the SIBDC data evidenced an association of need for more intense treatment, i.e. biological therapy (*p* = 0.02) and a trend for more intestinal surgery (*p* = 0.13) in our patient cohort. However, even nominally significant associations would not remain significant after Bonferroni correction, pointing to the need of large, well-characterized cohorts for studying the important questions of IBD genetic risk and disease course and treatment.

Our study has various strengths and limitations. We were able to use a large prospective cohort of well-characterized IBD patients to test associations of rs8005161 genotypes with disease course. Moreover, we were able to recruit patients with IBD and rare genotypes, thus enabling functional studies with individuals of each genotype for RhoA and cAMP activation. Limitations include the small sample size for all experiments, and lack of non-wildtype carriers of rs8005161 in the control population. Limited amount of patient material prohibited inclusion of the GPR65 inhibitor for RhoA tests. Future studies should also address expression levels of GPR65 RNA and start gene sequencing to test for polymorphisms associated with specific variants of the SNP.

## Conclusions

In summary, in the SIBDC patients, there is a trend for a more severe disease course for T allele carriers and a tendency towards a lower probability for anti-TNF treatment. In addition, we confirmed a significant association between the rare homozygote rs8005161 TT genotype and a diagnosis of UC. Our study did not identify biochemical changes in individuals with various genotypes of rs8005161, however we identified a lower activation of RhoA upon an acidic pH shift in IBD patients. Thus, pH sensors may be interesting new targets for pharmacological intervention in intestinal inflammation.

## Additional files

Additional file 1: **Figure S1.** Quality control for human peripheral blood mononuclear cell enrichment. PBMCs were separated by Ficoll density gradient centrifugation and purified using the EasySep Human Monocyte CD14 Enrichment Kit. Flow cytometry data analysis using antibodies allophycocyanin (APC)-labelled anti-CD14 and Pacific Blue (PB)-labelled anti-CD45 was performed to check cell purity. CD14+ cell purity after enrichment was > 85%. (DOCX 183 kb)

Additional file 2: **Figure S2.** Effect of pH on cAMP formation in (A) THP-1 cells and primary human CD14+ monocytes isolated from (B) healthy subjects. To confirm that the pH values were associated with the activation and inactivation of GPR65/cAMP G-protein mediated signalling a pH dose response curve was generated. THP-1 cells and primary CD14+ human monocytes (WT/CC) were starved at pH 7.6 for 2 h (to silence the receptor) and subsequently subjected to a pH shift for 10 min (pH 6.2 to 7.8 with 0.2 increments). The highest cAMP accumulation was observed at pH 6.4–6.8, whereas low cAMP concentrations were demonstrated at pH 7.6–7.8. (DOCX 674 kb)

Additional file 3: **Figure S3.** Effect of pH on RhoA activation in (A) THP-1 cells and (B) primary human CD14+ monocytes. Description of data: To confirm pH dependent RhoA activity, THP-1 cells and CD14+ monocytes were subjected to different pH (10 min) after a preliminary starvation step (2 h) at non-activating pH (pH 7.6) to silence the receptor. pH 6.6 elicited a significant increase in RhoA activation compared to pH 6.2, 7.4 and 7.6. GPR65/G_12/13_/RhoA signalling exhibits the highest activity at pH 6.6. (DOCX 65 kb)

Additional file 4: **Table S1.** Allele frequencies of SNP variants and allele association analysis within a population of IBD patients and healthy subjects. Allele frequencies of SNP variants GPR65, rs8005161 and rs3742704 and GALC rs1805078 for allele association analysis within a population of IBD patients and healthy subjects. (DOCX 43 kb)

Additional file 5: **Table S2.** Allele frequencies and biological phenotypes GPR65 SNP rs8005161 for patients from the SIBDC. Allele frequencies and biological phenotypes GPR65 SNP rs8005161 for patients from the Swiss IBD cohort (SIBDC). (DOCX 41 kb)

Additional file 6: **Figure S4.** Formation of cAMP in human CD14+ monocytes upon pH shift from pH 7.6 to pH 6.5. Description of data: (A) Baseline values pH 7.6 and (B) after 10 min at acidic pH (pH 6.5). Human CD14+ cells were obtained from IBD patients carrying either rs8005161 TT, CT or WT/CC genotype, and non-IBD control subjects - all WT/CC genotype. 10 μM of G protein-coupled receptor 65 (GPR65) antagonist was used (C, D). No significant differences between the genotypes were identified. These data are identical to Fig. 1 but presented without normalization. cAMP: cyclic adenosine monophosphate, IBD: inflammatory bowel disease, WT: Wild type. (DOCX 403 kb)

## Abbreviations

cAMP: Cyclic adenosine monophosphate
CD: Crohn’s disease
GALC: Galactosylceramidase
GPCR: G protein coupled receptor
GPR65: G protein coupled receptor 65
GWAS: Genome wide association studies
HBSS: Hank’s Balanced Salt Solution
IBD: Inflammatory bowel disease
NS: Not significant
OR: Odds ratio
PBS: Phosphate buffered saline
SIBDC: Swiss IBD Cohort Study
SNPs: Single nucleotide polymorphisms
TDAG8: T cell death associated gene 8
UC: Ulcerative colitis

## References

[1] Lardner A (2001). The effects of extracellular pH on immune function. J Leukoc Biol.

[2] Brokelman WJ, Lensvelt M, Borel Rinkes IH, Klinkenbijl JH, Reijnen MM (2011). Peritoneal changes due to laparoscopic surgery. Surg Endosc.

[3] Hanly EJ, Aurora AA, Shih SP, Fuentes JM, Marohn MR, De Maio A, Talamini MA (2007). Peritoneal acidosis mediates immunoprotection in laparoscopic surgery. Surgery.

[4] Martinez D, Vermeulen M, von Euw E, Sabatte J, Maggini J, Ceballos A, Trevani A, Nahmod K, Salamone G, Barrio M (2007). Extracellular acidosis triggers the maturation of human dendritic cells and the production of IL-12. J Immunol.

[5] Ishii S, Kihara Y, Shimizu T (2005). Identification of T cell death-associated gene 8 (TDAG8) as a novel acid sensing G-protein-coupled receptor. J Biol Chem.

[6] Ludwig MG, Vanek M, Guerini D, Gasser JA, Jones CE, Junker U, Hofstetter H, Wolf RM, Seuwen K (2003). Proton-sensing G-protein-coupled receptors. Nature.

[7] Mogi C, Nakakura T, Okajima F (2014). Role of extracellular proton-sensing OGR1 in regulation of insulin secretion and pancreatic beta-cell functions. Endocr J.

[8] Okajima F (2013). Regulation of inflammation by extracellular acidification and proton-sensing GPCRs. Cell Signal.

[9] Seuwen K, Ludwig MG, Wolf RM (2006). Receptors for protons or lipid messengers or both?. J Recept Signal Transduct Res.

[10] Mohebbi N, Benabbas C, Vidal S, Daryadel A, Bourgeois S, Velic A, Ludwig MG, Seuwen K, Wagner CA (2012). The proton-activated G protein coupled receptor OGR1 acutely regulates the activity of epithelial proton transport proteins. Cell Physiol Biochem.

[11] Saxena H, Deshpande DA, Tiegs BC, Yan H, Battafarano RJ, Burrows WM, Damera G, Panettieri RA, Dubose TD, An SS (2012). The GPCR OGR1 (GPR68) mediates diverse signalling and contraction of airway smooth muscle in response to small reductions in extracellular pH. Br J Pharmacol.

[12] Lassen KG, McKenzie CI, Mari M, Murano T, Begun J, Baxt LA, Goel G, Villablanca EJ, Kuo SY, Huang H (2016). Genetic coding variant in GPR65 alters lysosomal pH and links lysosomal dysfunction with colitis risk. Immunity.

[13] Franke A, McGovern DP, Barrett JC, Wang K, Radford-Smith GL, Ahmad T, Lees CW, Balschun T, Lee J, Roberts R (2010). Genome-wide meta-analysis increases to 71 the number of confirmed Crohn's disease susceptibility loci. Nat Genet.

[14] Jostins L, Ripke S, Weersma RK, Duerr RH, McGovern DP, Hui KY, Lee JC, Schumm LP, Sharma Y, Anderson CA (2012). Host-microbe interactions have shaped the genetic architecture of inflammatory bowel disease. Nature.

[15] Liu JZ, van Sommeren S, Huang H, Ng SC, Alberts R, Takahashi A, Ripke S, Lee JC, Jostins L, Shah T (2015). Association analyses identify 38 susceptibility loci for inflammatory bowel disease and highlight shared genetic risk across populations. Nat Genet.

[16] de Lange KM, Moutsianas L, Lee JC, Lamb CA, Luo Y, Kennedy NA, Jostins L, Rice DL, Gutierrez-Achury J, Ji SG (2017). Genome-wide association study implicates immune activation of multiple integrin genes in inflammatory bowel disease. Nat Genet.

[17] Lee JC, Espeli M, Anderson CA, Linterman MA, Pocock JM, Williams NJ, Roberts R, Viatte S, Fu B, Peshu N (2013). Human SNP links differential outcomes in inflammatory and infectious disease to a FOXO3-regulated pathway. Cell.

[18] Wang JQ, Kon J, Mogi C, Tobo M, Damirin A, Sato K, Komachi M, Malchinkhuu E, Murata N, Kimura T (2004). TDAG8 is a proton-sensing and psychosine-sensitive G-protein-coupled receptor. J Biol Chem.

[19] Altshuler D, Daly MJ, Lander ES (2008). Genetic mapping in human disease. Science.

[20] Attinkara R, Mwinyi J, Truninger K, Regula J, Gaj P, Rogler G, Kullak-Ublick GA, Eloranta JJ, Swiss IBDCSG (2012). Association of genetic variation in the NR1H4 gene, encoding the nuclear bile acid receptor FXR, with inflammatory bowel disease. BMC Res Notes.

[21] Scharl M, Mwinyi J, Fischbeck A, Leucht K, Eloranta JJ, Arikkat J, Pesch T, Kellermeier S, Mair A, Kullak-Ublick GA (2012). Crohn's disease-associated polymorphism within the PTPN2 gene affects muramyl-dipeptide-induced cytokine secretion and autophagy. Inflamm Bowel Dis.

[22] Pittet V, Juillerat P, Mottet C, Felley C, Ballabeni P, Burnand B, Michetti P, Vader JP, Swiss IBDCSG (2009). Cohort profile: the Swiss inflammatory bowel disease cohort study (SIBDCS). Int J Epidemiol.

[23] Storm N, Darnhofer-Patel B, van den Boom D, Rodi CP (2003). MALDI-TOF mass spectrometry-based SNP genotyping. Methods Mol Biol.

[24] Ihara Y, Kihara Y, Hamano F, Yanagida K, Morishita Y, Kunita A, Yamori T, Fukayama M, Aburatani H, Shimizu T (2010). The G protein-coupled receptor T-cell death-associated gene 8 (TDAG8) facilitates tumor development by serving as an extracellular pH sensor. Proc Natl Acad Sci U S A.

[25] Radu CG, Nijagal A, McLaughlin J, Wang L, Witte ON (2005). Differential proton sensitivity of related G protein-coupled receptors T cell death-associated gene 8 and G2A expressed in immune cells. Proc Natl Acad Sci U S A.

[26] Im DS, Heise CE, Nguyen T, O'Dowd BF, Lynch KR (2001). Identification of a molecular target of psychosine and its role in globoid cell formation. J Cell Biol.

[27] Onozawa Y, Fujita Y, Kuwabara H, Nagasaki M, Komai T, Oda T (2012). Activation of T cell death-associated gene 8 regulates the cytokine production of T cells and macrophages in vitro. Eur J Pharmacol.

[28] Choi J-W, Lee SY, Choi Y (1996). Identification of a putative G protein-coupled receptor induced during activation-induced apoptosis of T cells. Cell Immunol.

[29] Kyaw H, Zeng Z, Su K, Fan P, Shell BK, Carter KC, Li Y (1998). Cloning, characterization, and mapping of human homolog of mouse T-cell death-associated gene. DNA Cell Biol.

[30] Mogi C, Tobo M, Tomura H, Murata N, He XD, Sato K, Kimura T, Ishizuka T, Sasaki T, Sato T (2009). Involvement of proton-sensing TDAG8 in extracellular acidification-induced inhibition of Proinflammatory cytokine production in peritoneal macrophages. J Immunol.

[31] Serezani CH, Ballinger MN, Aronoff DM, Peters-Golden M (2008). Cyclic AMP: master regulator of innate immune cell function. Am J Respir Cell Mol Biol.

[32] Yan K, Gao LN, Cui YL, Zhang Y, Zhou X (2016). The cyclic AMP signaling pathway: exploring targets for successful drug discovery (review). Mol Med Rep.

[33] Raker VK, Becker C, Steinbrink K (2016). The cAMP pathway as therapeutic target in autoimmune and inflammatory diseases. Front Immunol.

[34] Aoki H, Mogi C, Okajima F (2014). Ionotropic and metabotropic proton-sensing receptors involved in airway inflammation in allergic asthma. Mediat Inflamm.

[35] Jin Y, Sato K, Tobo A, Mogi C, Tobo M, Murata N, Ishii S, Im DS, Okajima F (2014). Inhibition of interleukin-1beta production by extracellular acidification through the TDAG8/cAMP pathway in mouse microglia. J Neurochem.

[36] Nagasaka A, Mogi C, Ono H, Nishi T, Horii Y, Ohba Y, Sato K, Nakaya M, Okajima F, Kurose H (2017). The proton-sensing G protein-coupled receptor T-cell death-associated gene 8 (TDAG8) shows cardioprotective effects against myocardial infarction. Sci Rep.

[37] Onozawa Y, Komai T, Oda T (2011). Activation of T cell death-associated gene 8 attenuates inflammation by negatively regulating the function of inflammatory cells. Eur J Pharmacol.

[38] Lang P, Gesbert F, Delespine-Carmagnat M, Stancou R, Pouchelet M, Bertoglio J (1996). Protein kinase a phosphorylation of RhoA mediates the morphological and functional effects of cyclic AMP in cytotoxic lymphocytes. EMBO J.

[39] Laudanna C, Campbell JJ, Butcher EC (1997). Elevation of intracellular cAMP inhibits RhoA activation and integrin-dependent leukocyte adhesion induced by chemoattractants. J Biol Chem.

[40] Park PH, Huang H, McMullen MR, Bryan K, Nagy LE (2008). Activation of cyclic-AMP response element binding protein contributes to adiponectin-stimulated interleukin-10 expression in RAW 264.7 macrophages. J Leukoc Biol.

[41] Hatzelmann A, Morcillo EJ, Lungarella G, Adnot S, Sanjar S, Beume R, Schudt C, Tenor H (2010). The preclinical pharmacology of roflumilast--a selective, oral phosphodiesterase 4 inhibitor in development for chronic obstructive pulmonary disease. Pulm Pharmacol Ther.

[42] Lorenowicz MJ, Fernandez-Borja M, van Stalborch AM, van Sterkenburg MA, Hiemstra PS, Hordijk PL (2007). Microtubule dynamics and Rac-1 signaling independently regulate barrier function in lung epithelial cells. Am J Physiol Lung Cell Mol Physiol.

[43] Zimmerman NP, Kumar SN, Turner JR, Dwinell MB (2012). Cyclic AMP dysregulates intestinal epithelial cell restitution through PKA and RhoA. Inflamm Bowel Dis.

[44] Bustelo XR, Sauzeau V, Berenjeno IM (2007). GTP-binding proteins of the rho/Rac family: regulation, effectors and functions in vivo. Bioessays.

[45] Etienne-Manneville S, Hall A (2002). Rho GTPases in cell biology. Nature.

[46] Hodge RG, Ridley AJ (2016). Regulating rho GTPases and their regulators. Nat Rev Mol Cell Biol.

[47] Konigs V, Jennings R, Vogl T, Horsthemke M, Bachg AC, Xu Y, Grobe K, Brakebusch C, Schwab A, Bahler M (2014). Mouse macrophages completely lacking rho subfamily GTPases (RhoA, RhoB, and RhoC) have severe lamellipodial retraction defects, but robust chemotactic navigation and altered motility. J Biol Chem.

[48] Kozasa T, Hajicek N, Chow CR, Suzuki N (2011). Signalling mechanisms of RhoGTPase regulation by the heterotrimeric G proteins G12 and G13. J Biochem.

[49] Vogt S, Grosse R, Schultz G, Offermanns S (2003). Receptor-dependent RhoA activation in G12/G13-deficient cells: genetic evidence for an involvement of Gq/G11. J Biol Chem.

[50] Wettschureck N, Offermanns S (2005). Mammalian G proteins and their cell type specific functions. Physiol Rev.

[51] Worzfeld T, Wettschureck N, Offermanns S (2008). G(12)/G(13)-mediated signalling in mammalian physiology and disease. Trends Pharmacol Sci.

[52] Sugimoto N, Takuwa N, Okamoto H, Sakurada S, Takuwa Y (2003). Inhibitory and stimulatory regulation of Rac and cell motility by the G12/13-rho and Gi pathways integrated downstream of a single G protein-coupled sphingosine-1-phosphate receptor isoform. Mol Cell Biol.

[53] Parikh K, Zhou L, Somasundaram R, Fuhler GM, Deuring JJ, Blokzijl T, Regeling A, Kuipers EJ, Weersma RK, Nuij VJ (2014). Suppression of p21Rac signaling and increased innate immunity mediate remission in Crohn’s disease. Sci Transl Med.

[54] Cleynen I, Boucher G, Jostins L, Schumm LP, Zeissig S, Ahmad T, Andersen V, Andrews JM, Annese V, Brand S (2016). Inherited determinants of Crohn's disease and ulcerative colitis phenotypes: a genetic association study. Lancet.

[55] Ananthakrishnan AN, Huang H, Nguyen DD, Sauk J, Yajnik V, Xavier RJ (2014). Differential effect of genetic burden on disease phenotypes in Crohn's disease and ulcerative colitis: analysis of a north American cohort. Am J Gastroenterol.

