# Supplementary figures and images for: The impact of the rs8005161 polymorphism on G protein-coupled receptor GPR65 (TDAG8) pH-associated activation in intestinal inflammation
Source: BMC Gastroenterol. 2019 Jan 7;19:2. doi: 10.1186/s12876-018-0922-8 (PMC6323805; doi:10.1186/s12876-018-0922-8)

| **B**  **A** |
| --- |
| **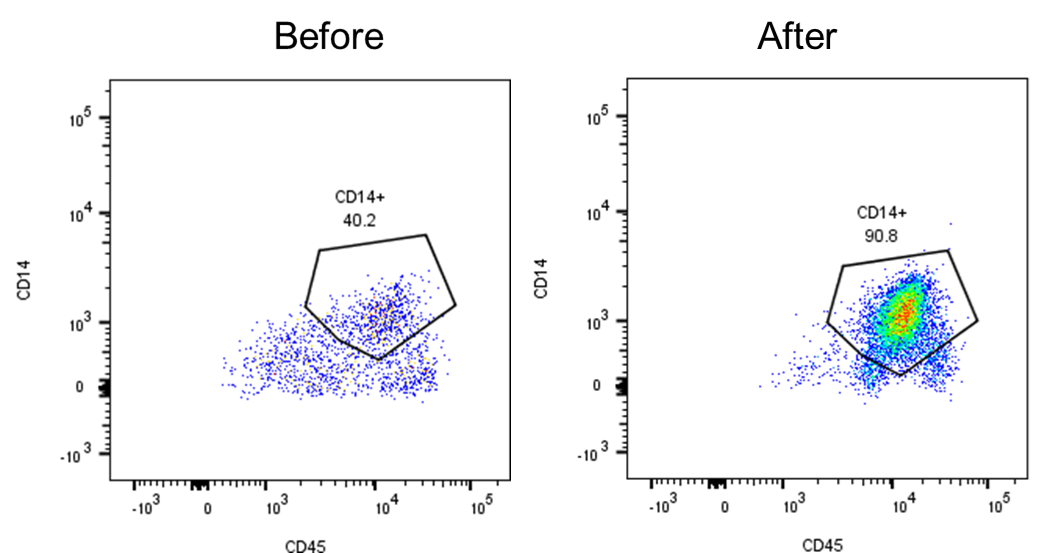** |

Supplement: Supplementary file 1 — Figure S1. Quality control for human peripheral blood mononuclear cell enrichment. PBMCs were separated by Ficoll density gradient centrifugation and purified using the EasySep Human Monocyte CD14 Enrichment Kit. Flow cytometry data analysis using antibodies allophycocyanin (APC)-labelled anti-CD14 and Pacific Blue (PB)-labelled anti-CD45 was performed to check cell purity. CD14+ cell purity after enrichment was > 85%. (DOCX 183 kb) [file 12876_2018_922_MOESM1_ESM.docx]

**B**

**A**

| **THP-1** | **CD14+** |
| --- | --- |
| **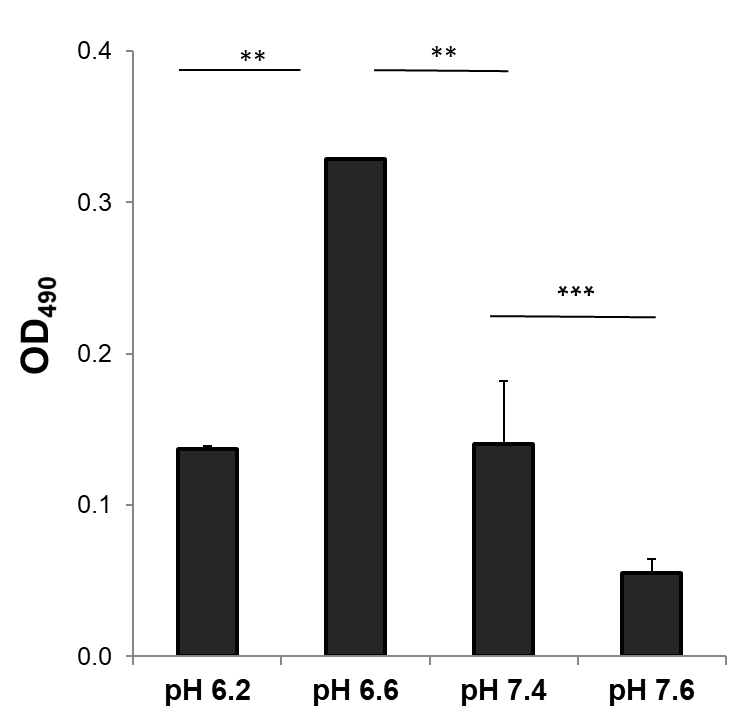** | **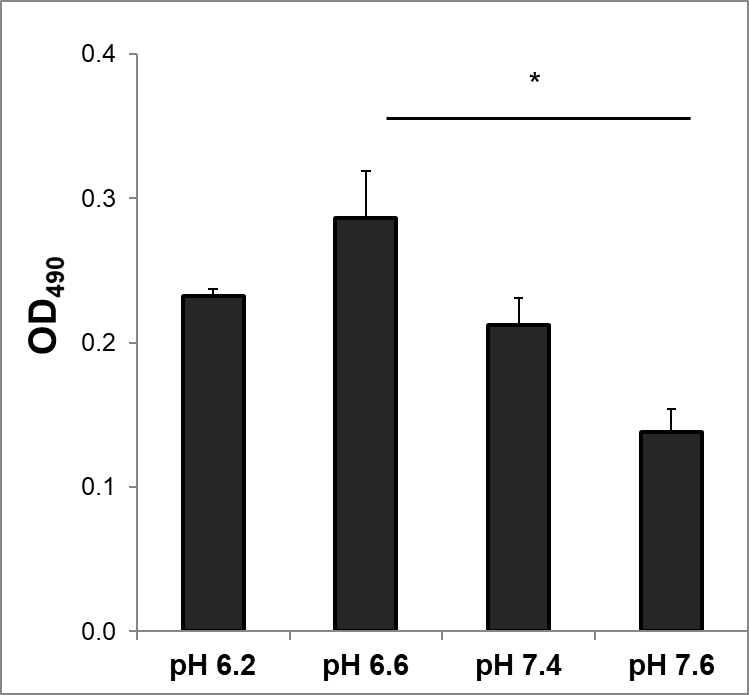** |
|  |  |

Supplement: Supplementary file 3 — Figure S3. Effect of pH on RhoA activation in (A) THP-1 cells and (B) primary human CD14+ monocytes. Description of data: To confirm pH dependent RhoA activity, THP-1 cells and CD14+ monocytes were subjected to different pH (10 min) after a preliminary starvation step (2 h) at non-activating pH (pH 7.6) to silence the receptor. pH 6.6 elicited a significant increase in RhoA activation compared to pH 6.2, 7.4 and 7.6. GPR65/G12/13/RhoA signalling exhibits the highest activity at pH 6.6. (DOCX 65 kb) [file 12876_2018_922_MOESM3_ESM.docx]

**
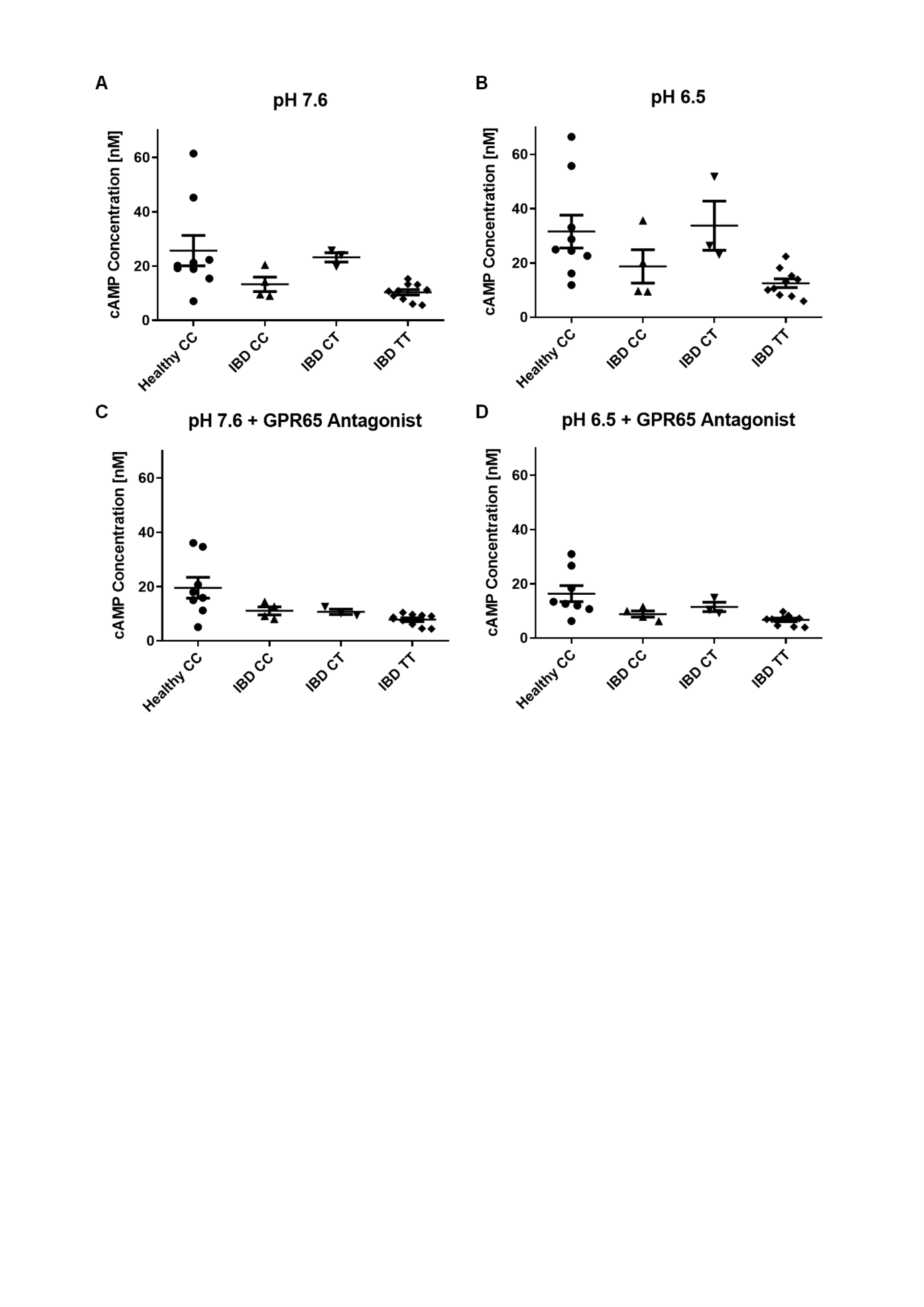
**

Supplement: Supplementary file 6 — Figure S4. Formation of cAMP in human CD14+ monocytes upon pH shift from pH 7.6 to pH 6.5. Description of data: (A) Baseline values pH 7.6 and (B) after 10 min at acidic pH (pH 6.5). Human CD14+ cells were obtained from IBD patients carrying either rs8005161 TT, CT or WT/CC genotype, and non-IBD control subjects - all WT/CC genotype. 10 μM of G protein-coupled receptor 65 (GPR65) antagonist was used (C, D). No significant differences between the genotypes were identified. These data are identical to Fig. 1 but presented without normalization. cAMP: cyclic adenosine monophosphate, IBD: inflammatory bowel disease, WT: Wild type. (DOCX 403 kb) [file 12876_2018_922_MOESM6_ESM.docx]
