# Supplementary material for: The impact of the rs8005161 polymorphism on G protein-coupled receptor GPR65 (TDAG8) pH-associated activation in intestinal inflammation
Source: BMC Gastroenterol. 2019 Jan 7;19:2. doi: 10.1186/s12876-018-0922-8 (PMC6323805; doi:10.1186/s12876-018-0922-8)
Supplement: Supplementary file 2 — Figure S2. Effect of pH on cAMP formation in (A) THP-1 cells and primary human CD14+ monocytes isolated from (B) healthy subjects. To confirm that the pH values were associated with the activation and inactivation of GPR65/cAMP G-protein mediated signalling a pH dose response curve was generated. THP-1 cells and primary CD14+ human monocytes (WT/CC) were starved at pH 7.6 for 2 h (to silence the receptor) and subsequently subjected to a pH shift for 10 min (pH 6.2 to 7.8 with 0.2 increments). The highest cAMP accumulation was observed at pH 6.4–6.8, whereas low cAMP concentrations were demonstrated at pH 7.6–7.8. (DOCX 674 kb) [file 12876_2018_922_MOESM2_ESM.docx]

**Figure S2.** **Effect of pH on cAMP formation in THP-1 cells (A-B) and primary human CD14+ monocytes isolated from healthy subjects (C-D).** Cells were treated in HBSS (pH 6.2 to 7.8). cAMP concentrations are normalized to non-activating pH 7.6 **(A, C).** Absolute values of cAMP formation are shown **(B, D).**
