# Supplementary material for: The impact of the rs8005161 polymorphism on G protein-coupled receptor GPR65 (TDAG8) pH-associated activation in intestinal inflammation
Source: BMC Gastroenterol. 2019 Jan 7;19:2. doi: 10.1186/s12876-018-0922-8 (PMC6323805; doi:10.1186/s12876-018-0922-8)
Supplement: Supplementary file 4 — Table S1. Allele frequencies of SNP variants and allele association analysis within a population of IBD patients and healthy subjects. Allele frequencies of SNP variants GPR65, rs8005161 and rs3742704 and GALC rs1805078 for allele association analysis within a population of IBD patients and healthy subjects. (DOCX 43 kb) [file 12876_2018_922_MOESM4_ESM.docx]

**Table S1. Allele frequencies of SNP variants and allele association analysis within a population of IBD patients and healthy subjects.**

| **Gene name** | **rs number** | **Alleles**  **Major/Minor** | | **Major allele** | **Minor allele** | **Minor allele frequency** | **p-value** | **OR** | | **CI** | |
| --- | --- | --- | --- | --- | --- | --- | --- | --- | --- | --- | --- |
| GPR65 | **rs8005161** | C/T | non-IBD | 939 | 105 | 10.05% |  |  |  | | |
|  |  |  | IBD | 632 | 92 | 12.7% | 0.09 | 1.3 | | | 0.97 –0.76 |
|  |  |  | UC | 241 | 41 | 14.5% | **0.041** | **1.5** | | | **1.04-2.24** |
|  |  |  | CD | 391 | 51 | 11.5% | 0.41 | 1.17 | | | 0.82-1.6 |
|  | **rs3742704** | A/C | non-IBD | 691 | 61 | 8.1% |  |  | | |  |
|  |  |  | IBD | 764 | 87 | 8.3% | 0.17 | 1.29 | | | 0,92-1,8 |
|  |  |  | UC | 221 | 23 | 9.4% | 0.51 | 1.18 | | | 0.7-1.92 |
|  |  |  | CD | 532 | 64 | 10.7% | 0.11 | 1.36 | | | 0.94-1.99 |
| GALC | **rs1805078** | G/A | non-IBD | 573 | 39 | 6.4% |  |  | | |  |
|  |  |  | IBD | 773 | 52 | 6.3% | 0.91 | 0.99 | | | 0.65-1.5 |
|  |  |  | UC | 247 | 21 | 7.8% | 0.47 | 1.25 | | | 0.72-2.2 |
|  |  |  | CD | 515 | 31 | 5.6% | 0.71 | 0.88 | | | 0.55-1.4 |

The total number of minor alleles was compared for IBD patients and healthy subjects (Fisher’s exact test. OR: odds ratio, CI: confidence interval, HWE: Hardy-Weinberg equilibrium, CD: Crohn’s disease, UC: ulcerative colitis).
