# Supplementary material for: The impact of the rs8005161 polymorphism on G protein-coupled receptor GPR65 (TDAG8) pH-associated activation in intestinal inflammation
Source: BMC Gastroenterol. 2019 Jan 7;19:2. doi: 10.1186/s12876-018-0922-8 (PMC6323805; doi:10.1186/s12876-018-0922-8)
Supplement: Supplementary file 5 — Table S2. Allele frequencies and biological phenotypes GPR65 SNP rs8005161 for patients from the SIBDC. Allele frequencies and biological phenotypes GPR65 SNP rs8005161 for patients from the Swiss IBD cohort (SIBDC). (DOCX 41 kb) [file 12876_2018_922_MOESM5_ESM.docx]

**Table S2. Allele frequencies and biological phenotypes GPR65 SNP rs8005161 for patients from the SIBDC.**

|  | rs8005161  CC | rs8005161  CT | rs8005161  TT | p-value (Fisher or Kruskal-Wallis) |
| --- | --- | --- | --- | --- |
| Gender  Male (n=1191)  Female (n=1109) | 953 (51.7%)  889 (48.3%) | 225 (52.3%)  205 (47.7%) | 13 (46.4%)  15 (53.6%) | 0.830 (n.s.) |
| Diagnosis  CD (n=1335)  UC / IC (n=965) | 1051 (57.1%)  791 (42.9%) | 269 (62.6%)  161 (37.4%) | 15 (53.6%)  13 (46.4%) | 0.102 (n.s.) |
| Age at diagnosis [years]  median, q25 – q75,  min – max,  n | 26.4, 19.1 – 36.6,  0.5 – 81.4,  n=1840 | 24.9, 18.2 – 35.9,  2.6 – 74.1,  n=429 | 27.6, 19.2 – 39.8,  7.5 – 77.5,  n=28 | 0.134 (n.s.) |
| Disease duration [years]  median, q25 – q75,  min – max,  n | 12.2, 7.3 – 20.5,  0.1 – 52.4,  n=1840 | 12.7, 7.6 – 21,  0.3 – 56.6,  n=429 | 7.9, 4.7 – 13.5,  2.1 – 31.7,  n=28 | **0.027** |
| Last BMI [kg /m^2^]  median, q25 – q75,  min – max,  n | 23.8, 21.1 – 26.7,  12.6 – 47.1,  n=1826 | 23.4, 20.7 – 26.1,  13.2 – 46.3,  n=426 | 22.7, 21.0 – 26.2,  18.2 – 33.7,  n=28 | 0.066 (n.s.) |
| Intestinal surgery  No (n=1620)  Yes (n=680) | 1311 (71.2%)  531 (28.8%) | 286 (66.5%)  144 (33.5%) | 23 (82.1%)  5 (17.9%) | 0.070 (n.s.) |
| Past or current therapy  with biologics  No (n=1107)  Yes (n=1193) | 909 (49.3%)  933 (50.7%) | 186 (43.3%)  244 (56.7%) | 12 (42.9%)  16 (57.1%) | 0.064 (n.s.) |

Significant phenotypes are indicated in bold. BMI: body mass index, IQR: interquartile range, CD: Crohn’s disease, UC: ulcerative colitis
